# General Therapeutics

**Published:** 1836

**Authors:** 


### Art. V.—General Therapeutics.

*General Therapeutics, or Principles of Medical Practice*, with tables of the chief remedial agents, and their preparation, and of the different poisons and their antidotes. By Robley Dunglison, M. D., etc. Philadelphia, Carey, Lea and Blanchard, 1836, pp. 580, 8 vo.

Few writers in our profession, have been more industrious than Professor Dunglison, and fewer still have sustained themselves equally as well in the course of so many practical publications. From the hasty perusal which we have given the above work, we are inclined to think that it possesses equal if not superior merit to any which have preceded it from the prolific pen of its author. The style of the Doctor is plain and simple and wholly divested of the useless ornament which marks the pages of so many of our modern writers.

We will not attempt, at present, to enter into an analysis of the book before us. This we will most probably do at a future period. At present we have only room enough to allow of a brief general notice of the work.

Unlike most writers our author has sent his work abroad without a preface, and of course without apologies or promises. He commences his book with a chapter on the general principles of Therapeutics, and closes it with a couple of copious indexes, leaving it for the reader to discover its merits or demerits by a perusal of the whole, a task which we have performed in a hasty manner. From what we have learned, however, we venture to say that few enquiring minds will rest satisfied with such a perusal of the volume. It shows the learning and research of its author on every page, and as an electic production, it will bear comparison with similar works, in any country. Unlike the works of Chapman and others, it does not enter upon a description of the different curative agents, with a list of diseases, which each is said to relieve, appended, but it takes up the different classes of medicine, and investigates them as a class, with the indications they are to fulfil, in the most philosophical manner, leaving it for the student or practitioner to select the particular medicine required, according to the dictates of his own judgement and nature of the disease. The student who opens the works of Eberle and others will find that a single medicine is frequently said to cure almost all the diseases to which flesh is heir. This only serves to perplex his mind without giving him any just notions of the department which he is about to study. We would advise our readers to purchase and peruse it for themselves.

